# Increasing Cropping System Diversity Balances Productivity, Profitability and Environmental Health

**DOI:** 10.1371/journal.pone.0047149

**Published:** 2012-10-10

**Authors:** Adam S. Davis, Jason D. Hill, Craig A. Chase, Ann M. Johanns, Matt Liebman

**Affiliations:** 1 United States Department of Agriculture/Agricultural Research Service, Global Change and Photosynthesis Research Unit, Urbana, Illinois, United States of America; 2 Department of Bioproducts and Biosystems Engineering, University of Minnesota, St. Paul, Minnesota, United States of America; 3 Leopold Center for Sustainable Agriculture, Iowa State University, Ames, Iowa, United States of America; 4 Department of Economics, Iowa State University Extension and Outreach, Osage, Iowa, United States of America; 5 Department of Agronomy, Iowa State University, Ames, Iowa, United States of America; New York State Museum, United States of America

## Abstract

Balancing productivity, profitability, and environmental health is a key challenge for agricultural sustainability. Most crop production systems in the United States are characterized by low species and management diversity, high use of fossil energy and agrichemicals, and large negative impacts on the environment. We hypothesized that cropping system diversification would promote ecosystem services that would supplement, and eventually displace, synthetic external inputs used to maintain crop productivity. To test this, we conducted a field study from 2003–2011 in Iowa that included three contrasting systems varying in length of crop sequence and inputs. We compared a conventionally managed 2-yr rotation (maize-soybean) that received fertilizers and herbicides at rates comparable to those used on nearby farms with two more diverse cropping systems: a 3-yr rotation (maize-soybean-small grain + red clover) and a 4-yr rotation (maize-soybean-small grain + alfalfa-alfalfa) managed with lower synthetic N fertilizer and herbicide inputs and periodic applications of cattle manure. Grain yields, mass of harvested products, and profit in the more diverse systems were similar to, or greater than, those in the conventional system, despite reductions of agrichemical inputs. Weeds were suppressed effectively in all systems, but freshwater toxicity of the more diverse systems was two orders of magnitude lower than in the conventional system. Results of our study indicate that more diverse cropping systems can use small amounts of synthetic agrichemical inputs as powerful tools with which to tune, rather than drive, agroecosystem performance, while meeting or exceeding the performance of less diverse systems.

## Introduction

One of the key challenges of the 21st century is developing ways of producing sufficient amounts of food while protecting both environmental quality and the economic well-being of rural communities [1], [2]. Over the last half century, conventional approaches to crop production have relied heavily on manufactured fertilizers and pesticides to increase yields, but they have also degraded water quality and posed threats to human health and wildlife [3]–[6]. Consequently, attention is now being directed toward the development of crop production systems with improved resource use efficiencies and more benign effects on the environment [1], [7]. Less attention has been paid to developing better methods of pest management, especially for weeds. Here we explore the potential benefits of diversifying cropping systems as a means of controlling weed population dynamics while simultaneously enhancing other desirable agroecosystem processes [8]. We focus on crop rotation, an approach to cropping system diversification whereby different species are placed in the same field at different times.

Rotation systems have been used for millennia to maintain soil fertility and productivity and to suppress pests, and can increase yields even in situations where substantial amounts of fertilizers and pesticides are applied [9], [10]. Rotation systems also foster spatial diversity, since different crops within the rotation sequence are typically grown in different fields on a farm in the same year. Diversification through crop rotation can be an especially useful strategy in farming systems that integrate crop and livestock production. The addition of forage crops, including turnips and clovers, to cereal-based systems in northwestern Europe and England in the 1600s and 1700s enhanced nitrogen supply through fixation by legumes, and increased nutrient cycling due to greater livestock density and manure production. These changes allowed the intensification of both crop and livestock production and increased yields substantially [11], [12]. Integrated crop–livestock systems remained widespread in northern Europe, England, and much of the humid, temperate regions of North America until the 1950s and 1960s, when increased availability of relatively low-cost synthetic fertilizers made mixed farming and nutrient recycling biologically unnecessary and specialized crop and livestock production more economically attractive. In recent years, there has been interest in reintegrating crop and livestock systems as a strategy for reducing reliance on fossil fuels, minimizing the use of increasingly expensive fertilizers, and limiting water pollution by nutrients, pathogens, and antibiotics [13], [14].

Weeds are a ubiquitous and recurrent problem in essentially all crop production systems, and chemicals applied for weed control dominate the world market for pesticides [15]. With the introduction of crop genotypes engineered to tolerate herbicides, especially glyphosate, and with the continuing availability of older, relatively inexpensive herbicides, such as atrazine, successful weed management in conventional crop production systems has been largely taken for granted since the mid-1990s. Now, however, with expanded recognition of herbicides as environmental contaminants [4] and the increasing prevalence of herbicide resistant weeds [16], there is an important need to develop weed management strategies that are less reliant on herbicides and that subject weeds to a wide range of stress and mortality factors [17]. We believe that cropping system diversification may play an important role in the development of such strategies.

Here, we report the results of a large-scale, long-term experiment examining the consequences of cropping system diversification on agronomic, economic, and environmental measures of system performance. The experiment was conducted during 2003–2011 in Boone County, Iowa, within the central U.S. maize production region, and comprised three contrasting cropping systems varying in length of crop sequence, levels of chemical inputs, and use of manure. We compared a conventionally managed 2-yr rotation (maize-soybean) that received fertilizers and herbicides at rates comparable to those used on surrounding commercial farms with two more diverse cropping systems: a 3-yr rotation (maize-soybean-small grain + red clover) and a 4-yr rotation (maize-soybean-small grain + alfalfa-alfalfa) managed with reduced N fertilizer and herbicide inputs and periodic applications of composted cattle manure. Triticale was used as the small grain crop in 2003–2005; oat was used in 2006–2011. The 2-yr rotation is typical of cash grain farming systems in the region, whereas the 3-yr and 4-yr rotations are representative of farming systems in the region that include livestock. Details of the experimental site, management practices, sampling procedures, and data analyses are provided in the online SI section (Text S1, Figure S1, Tables S1-S4).

A central hypothesis framing our study was that cropping system diversification would result in the development of ecosystem services over time that would supplement, or eventually displace, the role of synthetic external inputs in maintaining crop productivity and profitability. Based on this hypothesis, we predicted that input requirements of the more diverse systems would initially be similar to that of the less diverse system, but would increasingly diverge from the less diverse system over time as the systems matured. We also predicted that crop yields, weed suppression, and economic performance of the three systems would be similar throughout the study. Finally, we predicted that reduced requirements for external synthetic inputs for pest management would result in a lower toxicological profile of the more diverse systems compared to the less diverse system.

## Results

### Crop Yields and Net Profitability

**Figure 1:**
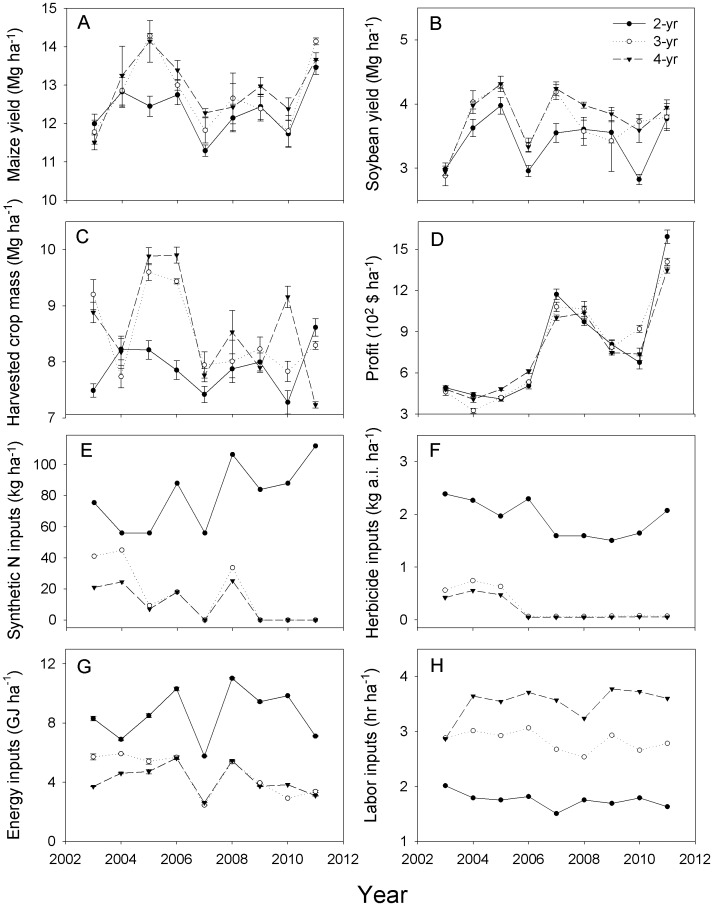
Cropping system performance over time. Annual performance of maize-soybean (2-yr), maize-soybean-small grain/red clover (3-yr), and maize-soybean-small grain/alfalfa-alfalfa (4-yr) cropping systems in Boone, IA, from 2003 to 2011. Performance metrics included: a) maize yield, b) soybean yield, c) rotation-level harvested crop mass, d) net returns to land and management, e) manufactured N fertilizer application rate, f) herbicide application rate, g) fossil energy use, and h) labor requirements. Symbols represent the mean ± SEM of four replicate experimental blocks (N = 36 per cropping system).

Cropping system diversification enhanced yields of maize and soybean grain and system-level harvested crop mass (grain, straw, and hay) while maintaining economic returns. The most parsimonious linear statistical models for each of these measures of system performance contained terms for main effects of *year* and *system*, but no interaction term (AIC_with interaction_ = 319; AIC _no interaction_ = 315). Over the 2003 to 2011 period, maize grain yield was on average 4% greater in the 3-yr and 4-yr rotations than in the 2-yr rotation (means for the 2-yr, 3-yr and 4-yr rotations are hereafter referred to as μ_2_, μ_3_ and μ_4_, respectively; μ_2_ = 12.3±0.1 Mg ha^−1^; μ_3_ = 12.7±0.2 Mg ha^−1^; μ_4_ = 12.9±0.2 Mg ha^−1^; pre-planned 1 d.f. contrast of *system*: F_1,7_ = 8, P = 0.03), and similar in the 3-yr and 4-yr rotations (Fig. 1a). Soybean grain yield during the same period was on average 9% greater in the 3-yr and 4-yr rotations than in the 2-yr rotation (μ_2_ = 3.4±0.07 Mg ha^−1^; μ_3_ = 3.8±0.08 Mg ha^−1^; μ_4_ = 3.8±0.08 Mg ha^−1^; F_1,7_ = 11.3, P = 0.01) and similar in the 3-yr and 4-yr rotations (Fig. 1b). Harvested crop mass, averaged over the various crop phases comprising each cropping system, followed a similar pattern to maize and soybean grain yields. Mean crop biomass for 2003 to 2011 was 8% greater in the 3-yr and 4-yr rotations than in the 2-yr rotation (μ_2_ = 7.9±0.08 Mg ha^−1^; μ_3_ = 8.5±0.1 Mg ha^−1^; μ_4_ = 8.6±0.2 Mg ha^−1^; *system*: t_6_ = 5.1, P = 0.002), and similar in the 3-yr and 4-yr rotations (Fig. 1c).

We examined system profitability by calculating net returns to land and management, which represent profits to a farm operation without accounting for costs of land (e.g., rent or mortgage payments), management time (e.g., marketing), and federal subsidies. Profitability was analyzed for two temporal periods. From 2003 to 2005, considered the “startup” phase for the study, there were no differences among cropping systems in net profit, either through an analysis of main effects of *system* (μ_2_ = $448±17 ha^−1^; μ_3_ = $402±17 ha^−1^; μ_4_ = $457±15 ha^−1^; F_2,6_ = 0.12, P = 0.89) or by pre-planned 1-d.f. contrasts (2-yr vs. 3-yr and 4-yr rotations: F_1,7_ = 0.10, P = 0.77) (Fig. 1d). From 2006 to 2011, the “established” phase of the study, there were again no differences among systems, either through main effects of *system* (μ_2_ = $953±36 ha^−1^; μ_3_ = $965±34 ha^−1^; μ_4_ = $913±26 ha^−1^; F_2,6_ = 0.62, P = 0.57) or by pre-planned 1-d.f. contrasts (2-yr vs. 3-yr and 4-yr rotations: F_1,7_ = 0.03, P = 0.86).

Stability of system performance over time, as measured through a comparison of variances for the various products of the system, was similar for maize grain yield (F_2,6_ = 2.4, P = 0.17), soybean grain yield (F_2,6_ = 0.95, P = 0.44) and net returns to land and management during the startup phase of the study, 2003 to 2005 (F_2,6_ = 0.05, P = 0.95). Two system products, harvested crop mass from 2003 to 2011 and profit during the established phase of the study, 2006 to 2011, showed considerable differences in system stability over time, but in contrasting ways. Variance in mean harvested crop mass was greater in the 3-yr and 4-yr rotations than in the 2-yr rotation (σ_2_^2^ = 0.27; σ_3_^2^ = 0.60; σ_4_^2^ = 0.95; F_1,7_ = 16, P = 0.005). Conversely, cropping system diversification was associated with lower variance in profit during the established phase of the study. Variance in profit from 2006 to 2011 was lower in the 3-yr and 4-yr rotations than in the 2-yr rotation (σ_2_^2^ = 1.5×10^5^; σ_3_^2^ = 8.1×10^3^; σ_4_^2^ = 6.3×10^3^; F_1,7_ = 16, P = 0.005).

### Agrichemical, Labor and Energy Inputs

Application rates of the primary agrichemicals used in this study, manufactured N fertilizer (F_2,14_ = 117, P<0.0001) and herbicides (F_2,14_ = 287, P<0.0001), both showed strong effects of cropping system. Manufactured N fertilizer applications were higher in the 2-yr rotation than in the 3-yr and 4-yr rotations (μ_2_ = 80±3 kg N ha^−1^; μ_3_ = 16±3 kg N ha^−1^; μ_4_ = 11±2 kg N ha^−1^; F_1,17_ = 16, P = 0.005), with the difference between systems increasing over the course of the study (F_2,14_ = 11.6, P = 0.001) (Fig. 1e). Herbicide application rates followed a similar pattern, with greater amounts of herbicide applied in the 2-yr rotation than in the 3-yr and 4-yr rotations (μ_2_ = 1.9±0.06 kg a.i. ha^−1^; μ_3_ = 0.26±0.05 kg a.i. ha^−1^; μ_4_ = 0.20±0.03 kg a.i. ha^−1^; F_1,17_ = 610, P<0.0001); differences among systems, however, did not increase over time (Fig. 1f).

Fossil energy use was strongly influenced by cropping system in both the startup (F_2,6_ = 94, P<0.0001) and established (F_2,6_ = 116, P<0.0001) phases of the study, with no difference in energy use between experimental phases (F_1,92_ = 0.39, P = 0.53) (Fig. 1g). From 2003 to 2011, inputs of energy were greater in the 2-yr rotation than in the 3-yr and 4-yr rotations (μ_2_ = 8.6±0.1 GJ ha^−1^; μ_3_ = 4.5±0.1 GJ ha^−1^; μ_4_ = 4.2±0.04 GJ ha^−1^; F_1,7_ = 55, P = 0.0001). The partial correlations between energy use in a given cropping system and energy use in the maize phase of that rotation, taking into account the amount of N fertilizer applied to maize, were 0.94, 0.81 and 0.70 in the 2-yr, 3-yr and 4-yr systems, respectively (SI, Table S5). This indicated that synthetic N fertilizer use in the maize phase of the various cropping systems drove energy use within the maize phase, which in turn drove energy use by a given cropping system.

Demand for labor differed among the three cropping systems in both the startup (F_2,4_ = 26, P = 0.005) and established (F_2,10_ = 299, P<0.0001) study phases, but followed a contrasting pattern to energy requirements (Fig. 1h). Labor inputs were more than 33% lower in the 2-yr rotation than in the 3-yr and 4-yr rotations from 2003 to 2005 (F_1,5_ = 35, P = 0.002) and from 2006 to 2011 (F_1,11_ = 59, P<0.0001). Overall, there was a strong negative correlation (r = −0.79, P<0.0001) between fossil energy and labor inputs over time in the three cropping systems.

### Divergent Weed Management Systems

**Figure 2:**
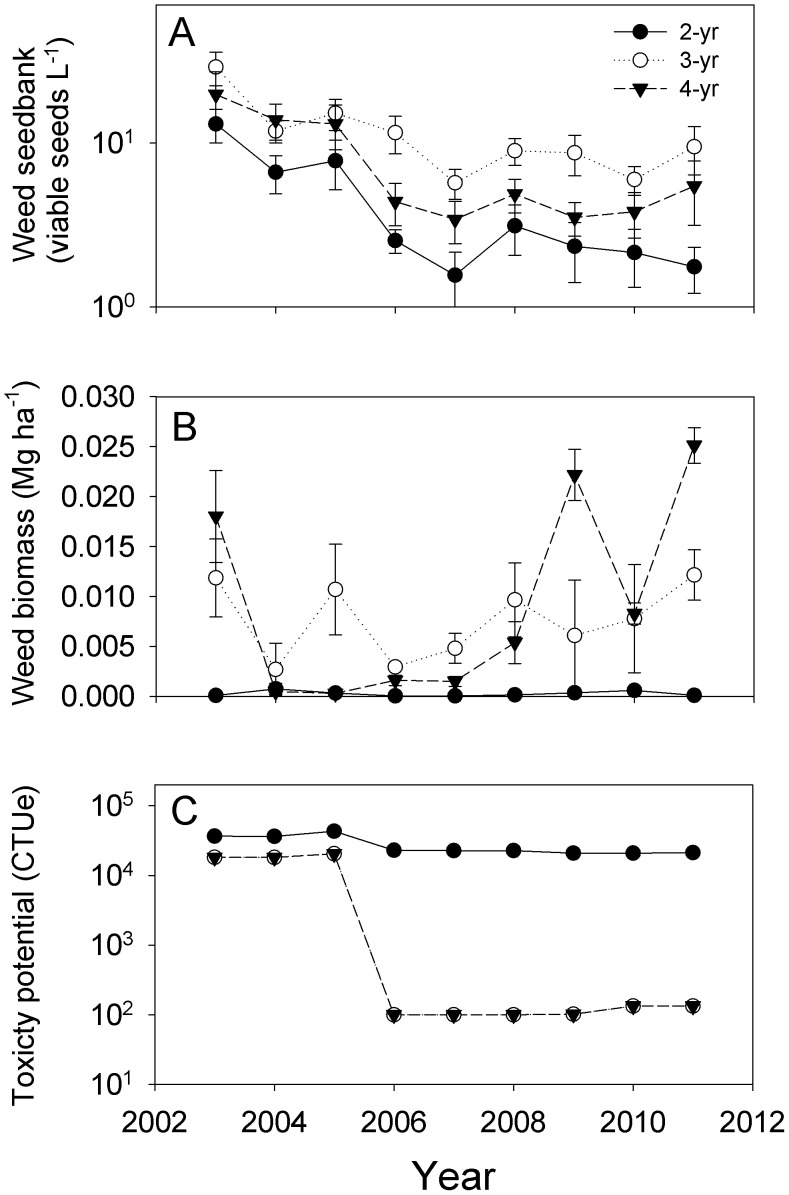
Divergent weed management systems. Weed management characteristics in maize-soybean (2-yr), maize-soybean-small grain/red clover (3-yr), and maize-soybean-small grain/alfalfa-alfalfa (4-yr) cropping systems in Boone, IA, from 2003 to 2011. Performance metrics included a) weed seed density in soil, b) weed aboveground biomass, and c) freshwater toxicity potential expressed in comparative toxic units (CTU_e_). Symbols represent the mean ± SEM of four replicate experimental blocks (N = 36 per cropping system).

Two lines of evidence indicate that weeds were managed effectively in all three cropping systems in both the ‘startup’ and ‘established’ phases, in spite of reducing herbicide use by 88% in the 3-yr and 4-yr rotations compared to the 2-yr rotation. First, weed seedbanks declined at an equal rate in all study systems (Fig. 2a). Selection among linear mixed effects regression models incorporating temporal autocorrelation among seedbank measurements over time supported different intercepts (*system*: F_2,6_ = 16.8, P = 0.0035) but did not support inclusion of a *year* by *system* interaction term (AIC_s_ = 182; AIC_s*y_ = 185), thus indicating a common slope (b_1_ = −0.18). For all three systems, the time to decline to 95% of the weed seedbank levels in 2003 was 16.6 years. Declines in weed seedbanks reflected a focus of management attention on the timing of weed management activities and herbicide choices in all three systems, as well as the increased number and diversity of stress and mortality factors present in the 3-yr and 4-yr rotations [8], [21]. Higher densities of weed seeds in the 3-yr and 4-yr rotations, as indicated by their greater intercept values than for the 2-yr rotation (Fig. 2a.), were the result of poorer weed control in the 3-yr and 4-yr rotations during the set-up of the experiment plots in 2002.

The second line of evidence concerns weed biomass, which was very low in all three cropping systems for the duration of the study (Fig. 2b), never exceeding 0.3% of harvested crop mass. Weed biomass was the same within a given crop phase, regardless of the cropping system in which it occurred (main effect of *system*: maize, F_2,6_ = 1.47; P = 0.30; soybean, F_2,6_ = 0.88; P = 0.46; small grain, F_1,3_ = 1.24; P = 0.31). There were differences in mean weed biomass among cropping systems (μ_2_ = 0.0003±0.00007 Mg ha^−1^; μ_3_ = 0.0076±0.0012 Mg ha^−1^; μ_4_ = 0.009±0.001 Mg ha^−1^; F_2,6_ = 12.7; P<0.007). These differences arose mainly due to the presence of a small grain phase in the 3-yr and 4-yr rotation crop sequences. Weed biomass did not differ between maize and soybean in any of the cropping systems (F_1,202_ = 2.1; P = 0.15), however weed biomass in the small grain phase of the 3-yr and 4-yr rotations was greater than weed biomass in the maize and soybean phases (F_1,206_ = 174; P<0.0001). In the 4-year system, weed biomass in alfalfa was intermediate between weed biomass levels in the maize/soybean and small grain phases.

Environmental toxicity, in relation to ecotoxicological profiles for herbicides used in this study (Fig. 2c), showed a strong effect of *system* (F_2,14_ = 1673, P<0.0001), with lower toxicity potential in the 3-yr and 4-yr rotations compared to the 2-yr rotation (*type*: F_1,17_ = 2691, P<0.0001). Ecotoxicity in the diversified and conventional systems diverged as the systems matured over time [*type* x *phase*: F_1,16_ = 7.4, P = 0.015], transitioning from a two-fold difference during 2003 to 2005 to a two hundred-fold difference in toxicity from 2006 to 2011 (Fig. 2c).

## Discussion

**Figure 3:**
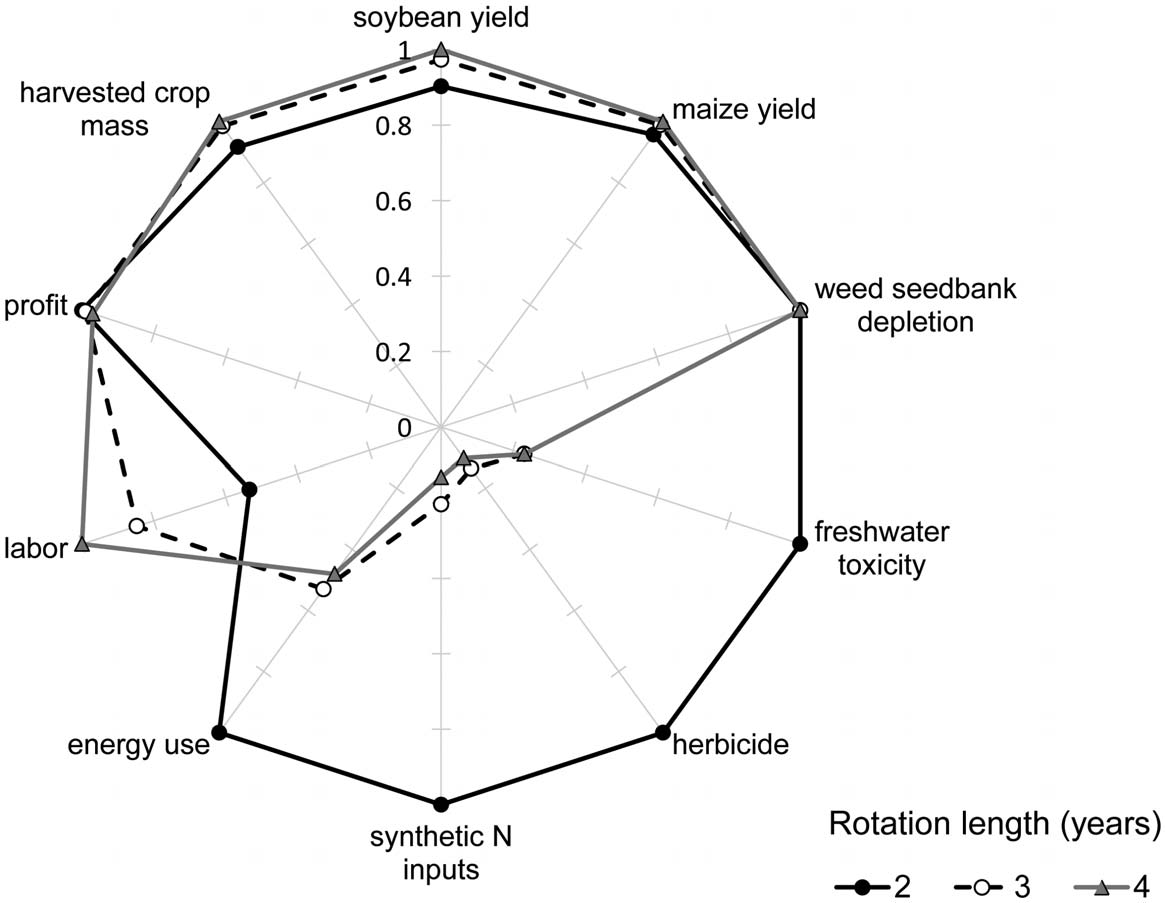
Multiple indicators of cropping system performance. Comparative long-term performance of maize-soybean (2-yr), maize-soybean-small grain/red clover (3-yr), and maize-soybean-small grain/alfalfa-alfalfa (4-yr) cropping systems in Boone, IA, averaged over the 2003–2011 study period. Variable means are normalized on a 0 to 1 scale, with 1 representing the cropping system with the largest absolute value for that variable (N = 36 per cropping system). Performance metrics included: maize and soybean yield, rotation-level harvested crop mass, net returns to land and management, manufactured N fertilizer and herbicide application rate, fossil energy use, labor requirements, freshwater toxicity potential and weed seedbank decline (measured as exponential decay constant).

Our results support the hypothesis that the development of ecosystem services over time in more diverse cropping rotations increasingly displaces the need for external synthetic inputs to maintain crop productivity. From 2003 to 2011, as predicted, the desired products (crop yield, weed suppression, and economic performance) of the more diverse and less diverse cropping rotations were similar, whereas external inputs and environmental impacts differed greatly among the systems (Fig. 3). Comparing these metrics of system performance by experimental phase (initial three years of system establishment versus the following six years) confirmed our prediction that system inputs and environmental impacts would diverge over time, whereas yield and profit would remain similar among more diverse and less diverse rotations. In the more diverse rotations, small amounts of synthetic agrichemical inputs thus served as powerful tools with which to tune, rather than drive, agroecosystem performance.

Grain production in the U.S. is dominated by short rotation systems designed to maximize grain yield and profit. These are important goals but represent only a portion of the many ecosystem services that managed lands may provide [18] and that should be considered when evaluating alternative production systems [1], [19]. We believe that these functions are complementary, rather than competing, considerations for agroecosystem design. The results of this study demonstrate that more rotationally diverse cropping systems may be optimized in multiple dimensions, leveraging small agrichemical inputs with biological synergies arising from enhanced diversity of crop species and management tactics.

An example of the synergizing effects of cropping system diversification can be found in weed management in the 3-yr and 4-yr rotations. Weeds were suppressed as effectively in these systems as in the 2-yr rotation, with declining soil seedbanks and negligible weed biomass, yet herbicide inputs in the 3-yr and 4-yr rotation plots were 6 to 10 times lower, and freshwater toxicity 200 times lower, than in the 2-yr rotation. Improved efficiency and environmental sustainability of weed management in the 3-yr and 4-yr rotations resulted from integrating multiple, complementary tactics in an ecological weed management framework [8], [20]. Mounting evidence for unintended effects resulting from heavy reliance on herbicides highlights the need to re-think the role of herbicides in weed management. Non-target impacts of herbicides include reproductive abnormalities and mortality in vertebrates [5], [21]–[23] and potential for diminished non-crop nectar resources for key pollinator species [17], [24], [25]. Herbicide overuse has also resulted in widespread, accelerating evolution of weed genotypes resistant to one or more modes of herbicide action [26], [27]. Our data indicate that, in the context of a cropping system with weed suppressive characteristics, small herbicide inputs may contribute to a diverse suite of tactics that cumulatively provide effective, reliable, and more durable weed management.

The diversity-productivity-stability relationship has long been a key theme in ecology [28], [29]. Recently, it has been applied in the context of bioenergy crop production to describe increases in biomass and ecosystem services, such as C sequestration, associated with increasing species diversity in polycultures of bioenergy feedstock crop species [30]. Our work supports the application of this concept to cropping systems more broadly. Future gains in more diverse systems may depend upon the application of ecological principles surrounding this relationship to cropping system design [31], [32]. Cropping system diversification in this study included both crop species and management practices. In contrast to the 2-yr rotation, with two species, both of the 3-yr and 4-yr rotations included four crop species. In the 4-yr rotation, further temporal diversification was achieved by including a perennial-only crop phase (alfalfa hay) for one quarter of the rotation sequence. Our results showed productivity gains associated with greater diversity in system-level harvested crop mass and maize and soybean seed yields. We also observed increased stability of profit, with similar long-term means, in the 3-yr and 4-yr rotations compared to the 2-yr rotation.

Similar profits were attained through different pathways in the 3-yr and 4-yr rotations and the 2-yr rotation (Fig. 3). Increased labor, information intensive management and ecosystem services arising from increased biological N fixation (via the clover and alfalfa crops) and contrasting crop phenologies and competitive abilities were substituted in 3-yr and 4-yr rotations for the higher inputs of manufactured N, herbicides and energy from fossil fuels driving the 2-yr rotation. Energy use in maize drove differences among the cropping systems, and manufactured N inputs to maize contributed most strongly to energy balances for this crop. The high sensitivity of agricultural energy use to N fertilizer inputs provides a high-priority target for the redesign of cropping systems for increased sustainability.

Reintegration of crop and livestock production, as represented by the forage legumes and manure applications present in the more diverse systems, is not simply another aspect of cropping system diversification. Instead, it embodies an important principle in sustainable agriculture: system boundaries should be drawn to minimize externalities. Animal manure is produced regardless of whether feed grains are shipped to centralized concentrated animal feeding operations, or produced within integrated crop-livestock farming operations. In the former case, the manure may become a waste product and water pollutant if quantities exceed available land area for field application [33], whereas in the latter case, it contributes directly to crop nutrient requirements, improves soil quality, and reduces fossil fuel subsidies associated with grain transport and external N fertilizer inputs [14].

Substantial improvements in the environmental sustainability of agriculture are achievable now, without sacrificing food production or farmer livelihoods. When agrichemical inputs are completely eliminated, yield gaps may exist between conventional and alternative systems [19]. However, such yield gaps may be overcome through the strategic application of very low inputs of agrichemicals in the context of more diverse cropping systems. Although maize is grown less frequently in the 3-yr and 4-yr rotations than in the 2-yr rotation, this will not compromise the ability of such systems to contribute to the global food supply, given the relatively low contribution of maize and soybean production to direct human consumption and the ability of livestock to consume small grains and forages [34]. Through a balanced portfolio approach to agricultural sustainability, cropping system performance can be optimized in multiple dimensions, including food and biomass production, profit, energy use, pest management, and environmental impacts.

## Materials and Methods

### Site Details and Agronomic Management

To investigate the relative performance of conventional and more diverse cropping systems, we conducted a 9-hectare experiment at the Iowa State University Marsden Farm (Figure S1), in Boone County, IA (42°01′ N; 93°47′ W; 333 m above sea level). The experiment site lies within a region of intensive rain-fed maize and soybean production and is surrounded by farms with high levels of productivity. Soils at the site are deep, fertile Mollisols. The experimental cropping system treatments included a conventionally managed 2-yr rotation (maize/soybean) that received agrichemicals at rates comparable to those used on commercial farms in the region, and more diverse cropping systems – a 3-yr rotation (maize/soybean/small grain + red clover green manure) and a 4-yr rotation (maize/soybean/small grain + alfalfa/alfalfa hay) – managed with reduced N fertilizer and herbicide inputs.

The entire site was planted with oat in 2001 and the cropping systems experiment was established in 2002 using a randomized complete block design with each crop phase of each rotation system present every year in four replicate blocks. Plots were 18 m x 85 m and managed with conventional farm machinery. Spring triticale was used as the small grain in 2003–2005, whereas oat was used in 2006–2010. Synthetic fertilizers were applied in the 2-yr rotation at conventional rates based on soil tests. In the 3-yr and 4-yr rotations, composted cattle manure was applied before maize production at a mean dry matter rate of 8.3 Mg ha^−1^ and substantial amounts of N were added through fixation by red clover and alfalfa [35], [36], [37]. Manure and legume N-fixation in the 3-yr and 4-yr rotations were supplemented with synthetic fertilizers based on soil tests, including the late-spring soil nitrate test for maize production [38]. Weed management in the 2-yr rotation was based largely on herbicides applied at conventional rates. In the 3-yr and 4-yr rotations, herbicides were applied in 38-cm-wide bands in maize and soybean and inter-row zones were cultivated; no herbicides were applied in small grain and forage legume crops. Choices of post-emergence herbicides used in each of the systems were made based on the identities, densities, and sizes of weed species observed in the plots. Other details of the farming practices used in the different cropping systems are described in Liebman et al. [39] and in the online SI materials (Text S1). Sampling procedures for determining crop yields, weed biomass and weed seed densities in soil are also described in the online SI materials (Text S1).

### Energy and Economic Analyses

Energy inputs were divided into five categories: seed, fertilizer, pesticides, fuel for field operations, and propane and electricity used for drying maize grain after harvest. Data were obtained from logs describing all field operations, material inputs, and crop moisture characteristics for the experimental plots during the study period. Economic analyses measured performance characteristics of whole rotation systems under contrasting management strategies. We evaluated net returns to land and management on a unit land area basis, with land units divided in two equal portions for maize and soybean in the 2-yr rotation; three equal portions for maize, soybean, and small grains with red clover in the 3-yr rotation; and four equal portions for maize, soybean, small grains with alfalfa, and alfalfa in the 4-yr rotation. Net returns to land and management represented returns to a farm operation calculated without accounting for costs of land (e.g., rent or mortgage payments), management time (e.g., marketing), or possible federal subsidies. Data sources and assumptions for the energy and economic analyses are shown in the online SI materials.

### Ecotoxicological Calculations

Freshwater ecotoxicity of pesticide use was estimated using the USEtox model [40]–[42]. Characterization factors (CFs) of ecotoxicity potential for active ingredients included transport to freshwater via surface water, soil, and air. CFs were available for eight of ten active ingredients applied in the three rotations. The two active ingredients for which CFs were unavailable are not of particular concern for freshwater ecotoxicity due either to their low toxicity (mesotrione) or low infiltration and persistence in freshwaters (lactofen) [43].

### Statistical Analyses

The experiment was arranged in a randomized complete block design, with all entry points of the three crop rotations (i.e. all crops within each of the rotations) represented in four replicate blocks in each year of the study, for a total of 36 plots. Cropping system effects in time series data were analyzed using hierarchical linear mixed effects repeated measures models, modeling temporally correlated errors with an ARMA (auto-regressive moving average) correlation structure in the *nlme* package of R v.2.14.1 [44], [45]. Fixed effects included *cropping system* and *experimental phase* (startup = 2003 to 2005; established = 2006 to 2011), and random effects included *replicate block* nested within *cropping system* and *year*. Partial correlations were estimated using the *corpcor* package in R v.2.14.1. In contrast to data for quantitative observations (e.g. crop yield or weed biomass) that varied by replicate block and year, data for input variables, such as synthetic fertilizer or herbicides and associated environmental toxicity metrics, did not vary among blocks for a particular rotation entry point in a given year, but did vary among years. Therefore, site-year was treated as the source of experimental replication for these latter variables in our statistical tests for effects of *cropping system* and *experimental phase*. This led to contrasting degrees of freedom in reported F-tests for these two data types. Finally, for variables with non-constant variance among cropping systems over time (crop biomass and profit), we used the ‘varIdent’ variance function within the *nlme* package to explicitly model differences in variances among cropping systems for these variables within our mixed effects models.

## Supporting Information

Figure S1 **Aerial view of Marsden Farm study, Boone IA.** Crop abbreviations: m = maize, sb = soybean, g = small grain, a = alfalfa.
(TIF)

Table S1 **Mean monthly air temperature and total monthly precipitation during the 2003–2011 growing seasons, and long-term temperature and precipitation averages.** Data were collected about 1 km from the experimental site in Boone Co., IA.
(DOCX)

Table S2 **Crop identities and seeding rates in 2003–2011.**
(DOCX)

Table S3 **Macronutrients applied in manufactured fertilizers, herbicide adjuvants, and manure in 2003–2011.** Manufactured N, P, and K fertilizers were applied at rates that varied among years and rotations in response to soil test results. Manure was applied at a rate of 15.7 Mg ha^−1^ in maize phases of the 3-year and 4-year rotation systems, but moisture and nutrient concentrations varied among years, resulting in variable rates of macronutrient additions.
(DOCX)

Table S4 **Herbicide applications in 2003–2011 to maize and soybean in the three rotation systems.** No herbicides were used for triticale, oat, red clover, and alfalfa grown within the 3-yr and 4-yr systems. Reported application rates reflect the effect of banding of herbicides over crop rows in the 3-yr and 4-yr systems.
(DOCX)

Table S5 **Simple and partial correlations between energy use within a given crop phase and mean rotation energy use and between energy use within a given crop phase and N fertilizer application rates.**
(DOCX)

Text S1 **Detailed description of experimental site, management practices, scientific methods and statistical approach.**
(DOCX)
