# Supplementary figures and images for: Increasing Cropping System Diversity Balances Productivity, Profitability and Environmental Health
Source: PLoS One. 2012 Oct 10;7(10):e47149. doi: 10.1371/journal.pone.0047149 (PMC3468434; doi:10.1371/journal.pone.0047149)

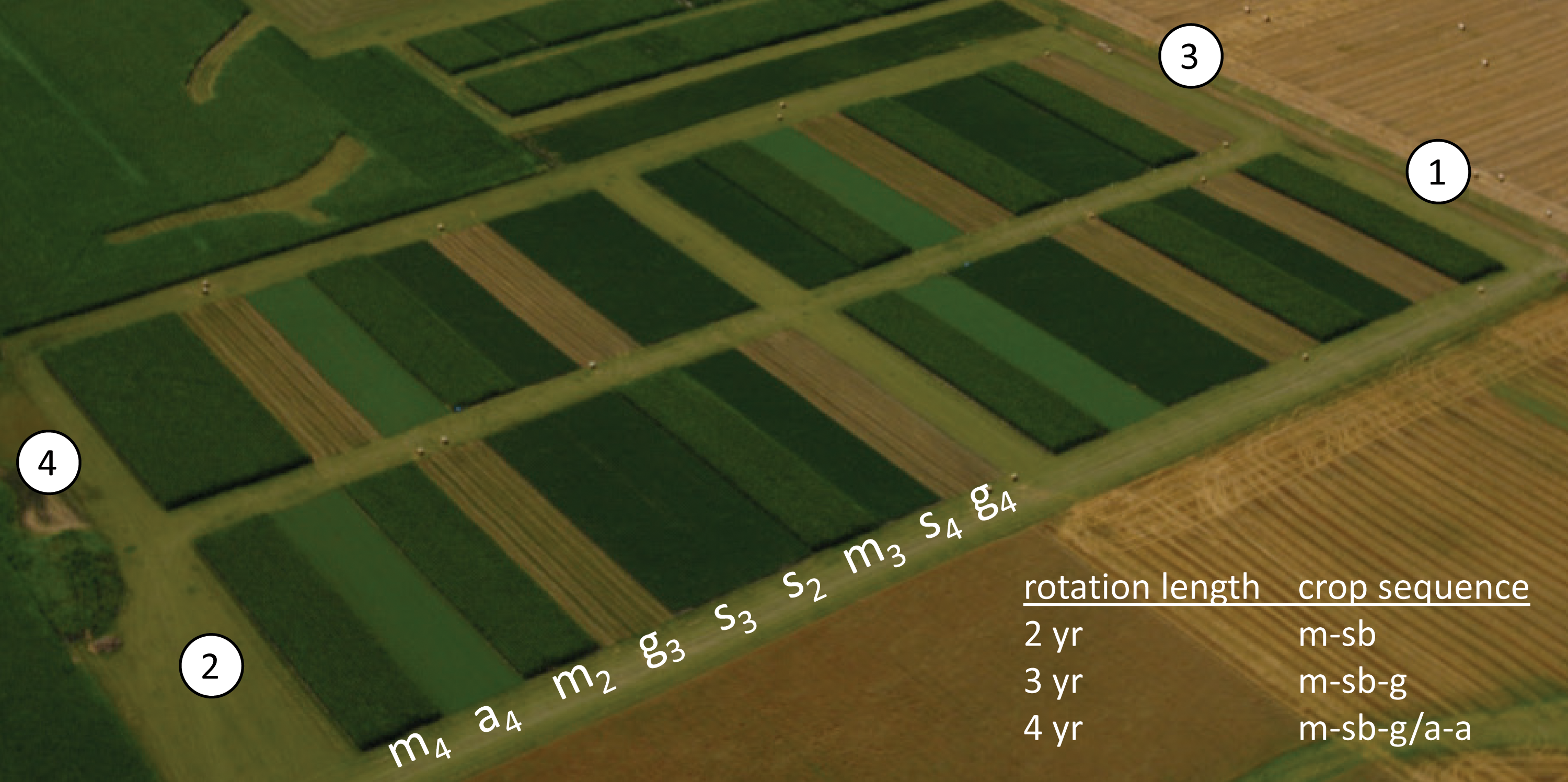

Supplement: Figure S1 — Aerial view of Marsden Farm study, Boone IA. Crop abbreviations: m = maize, sb = soybean, g = small grain, a = alfalfa. (TIF) [file pone.0047149.s001.tif]
