# Supplementary material for: Increasing Cropping System Diversity Balances Productivity, Profitability and Environmental Health
Source: PLoS One. 2012 Oct 10;7(10):e47149. doi: 10.1371/journal.pone.0047149 (PMC3468434; doi:10.1371/journal.pone.0047149)
Supplement: Table S1 — Mean monthly air temperature and total monthly precipitation during the 2003–2011 growing seasons, and long-term temperature and precipitation averages. Data were collected about 1 km from the experimental site in Boone Co., IA. (DOCX) [file pone.0047149.s002.docx]

**Table S1.** Mean monthly air temperature and total monthly precipitation during the 2003-2011 growing seasons (April – November), and long-term temperature and precipitation averages.

| **Year** | **Mean air temperature^a^ (°C)** | **Total precipitation (mm)** |
| --- | --- | --- |
| 2003 | 14.9 | 790 |
| 2004 | 15.0 | 697 |
| 2005 | 15.9 | 748 |
| 2006 | 15.6 | 777 |
| 2007 | 16.4 | 839 |
| 2008 | 15.2 | 1145 |
| 2009 | 14.8 | 755 |
| 2010 | 16.5 | 1165 |
| 2011 | 15.2 | 701 |
| 50-yr mean | 15.5 | 725 |

^a^ Data were collected about 1 km from the experimental site in Boone Co., IA.
