# Supplementary material for: Increasing Cropping System Diversity Balances Productivity, Profitability and Environmental Health
Source: PLoS One. 2012 Oct 10;7(10):e47149. doi: 10.1371/journal.pone.0047149 (PMC3468434; doi:10.1371/journal.pone.0047149)
Supplement: Table S2 — Crop identities and seeding rates in 2003–2011. (DOCX) [file pone.0047149.s003.docx]

**Table S2.** Crop identities and seeding rates in 2003-2011.

| **Year** | **Crop** | **Rotation** | **Hybrid or cultivar** | **Seeding density (no ha^-1^)** | **Seeding mass (kg ha^-1^)** |
| --- | --- | --- | --- | --- | --- |
| 2003 | Maize | 2-yr | Golden Harvest 8562 | 79,040 | na |
| 2003 | Maize | 3-yr | Golden Harvest 8562 | 79,040 | na |
| 2003 | Maize | 4-yr | Golden Harvest 8562 | 79,040 | na |
| 2003 | Soybean | 2-yr | Asgrow 2869 | 387,340 | na |
| 2003 | Soybean | 3-yr | Asgrow 2869 | 387,340 | na |
| 2003 | Soybean | 4-yr | Asgrow 2869 | 387,340 | na |
| 2003 | Triticale | 3-yr | Trical 37812 | na | 110 |
| 2003 | Triticale | 4-yr | Trical 37812 | na | 110 |
| 2003 | Red clover | 3-yr | Cherokee | na | 13 |
| 2003 | Alfalfa | 4-yr | Dekalb 3720 | na | 17 |
| 2004 | Maize | 2-yr | Golden Harvest 8562 | 79,040 | na |
| 2004 | Maize | 3-yr | Golden Harvest 8562 | 79,040 | na |
| 2004 | Maize | 4-yr | Golden Harvest 8562 | 79,040 | na |
| 2004 | Soybean | 2-yr | Asgrow 2869 | 387,340 | na |
| 2004 | Soybean | 3-yr | Asgrow 2869 | 387,340 | na |
| 2004 | Soybean | 4-yr | Asgrow 2869 | 387,340 | na |
| 2004 | Triticale | 3-yr | Trical 37812 | na | 122 |
| 2004 | Triticale | 4-yr | Trical 37812 | na | 122 |
| 2004 | Red clover | 3-yr | Cherokee | na | 13 |
| 2004 | Alfalfa | 4-yr | Dekalb 3720 | na | 17 |
| 2005 | Maize | 2-yr | Agrigold 6395 | 79,530 | na |
| 2005 | Maize | 3-yr | Agrigold 6395 | 79,530 | na |
| 2005 | Maize | 4-yr | Agrigold 6395 | 79,530 | na |
| 2005 | Soybean | 2-yr | Asgrow 2869 | 387,340 | na |
| 2005 | Soybean | 3-yr | Asgrow 2869 | 387,340 | na |
| 2005 | Soybean | 4-yr | Asgrow 2869 | 387,340 | na |
| 2005 | Triticale | 3-yr | Trical 37812 | na | 122 |
| 2005 | Triticale | 4-yr | Trical 37812 | na | 122 |
| 2005 | Red clover | 3-yr | Cherokee | na | 13 |
| 2005 | Alfalfa | 4-yr | Farm Science Genetics 300LH | na | 17 |
| 2006 | Maize | 2-yr | Agrigold 6395 | 79,530 | na |
| 2006 | Maize | 3-yr | Agrigold 6395 | 79,530 | na |
| 2006 | Maize | 4-yr | Agrigold 6395 | 79,530 | na |
| 2006 | Soybean | 2-yr | Kruger 287RR | 408,850 | na |
| 2006 | Soybean | 3-yr | Kruger 2918 | 408,850 | na |
| 2006 | Soybean | 4-yr | Kruger 2918 | 408,850 | na |
| 2006 | Oat | 3-yr | IN09201 | na | 56 |
| 2006 | Oat | 4-yr | IN09201 | na | 56 |
| 2006 | Red clover | 3-yr | Cherokee | na | 13 |
| 2006 | Alfalfa | 4-yr | Farm Science Genetics 400LH | na | 17 |
| 2007 | Maize | 2-yr | Agrigold 6395 | 79,530 | na |
| 2007 | Maize | 3-yr | Agrigold 6395 | 79,530 | na |
| 2007 | Maize | 4-yr | Agrigold 6395 | 79,530 | na |
| 2007 | Soybean | 2-yr | Kruger 287RR | 400,140 | na |
| 2007 | Soybean | 3-yr | Kruger 2918 | 400,140 | na |
| 2007 | Soybean | 4-yr | Kruger 2918 | 400,140 | na |
| 2007 | Oat | 3-yr | IN09201 | na | 62 |
| 2007 | Oat | 4-yr | IN09201 | na | 62 |
| 2007 | Red clover | 3-yr | Duration | na | 13 |
| 2007 | Alfalfa | 4-yr | Farm Science Genetics 400LH | na | 17 |
| 2008 | Maize | 2-yr | Agrigold 6395 BtRW | 79,530 | na |
| 2008 | Maize | 3-yr | Agrigold 6395 | 79,530 | na |
| 2008 | Maize | 4-yr | Agrigold 6395 | 79,530 | na |
| 2008 | Soybean | 2-yr | Kruger 287RR | 382,850 | na |
| 2008 | Soybean | 3-yr | Kruger 2918 | 382,850 | na |
| 2008 | Soybean | 4-yr | Kruger 2918 | 382,850 | na |
| 2008 | Oat | 3-yr | IN09201 | na | 83 |
| 2008 | Oat | 4-yr | IN09201 | na | 83 |
| 2008 | Red clover | 3-yr | Cherokee | na | 13 |
| 2008 | Alfalfa | 4-yr | Farm Science Genetics 400LH | na | 17 |
| 2009 | Maize | 2-yr | Agrigold 6395 BtRW | 79,530 | na |
| 2009 | Maize | 3-yr | Agrigold 6395 | 79,530 | na |
| 2009 | Maize | 4-yr | Agrigold 6395 | 79,530 | na |
| 2009 | Soybean | 2-yr | Kruger 287RR | 395,200 | na |
| 2009 | Soybean | 3-yr | Kruger 2918 | 395,200 | na |
| 2009 | Soybean | 4-yr | Kruger 2918 | 395,200 | na |
| 2009 | Oat | 3-yr | IN09201 | na | 78 |
| 2009 | Oat | 4-yr | IN09201 | na | 78 |
| 2009 | Red clover | 3-yr | Duration | na | 13 |
| 2009 | Alfalfa | 4-yr | Freedom LH | na | 17 |
| 2010 | Maize | 2-yr | Agrigold 6395 BtRW | 79,530 | na |
| 2010 | Maize | 3-yr | Agrigold 6395 | 79,530 | na |
| 2010 | Maize | 4-yr | Agrigold 6395 | 79,530 | na |
| 2010 | Soybean | 2-yr | Kruger 287RR | 395,940 | na |
| 2010 | Soybean | 3-yr | Kruger 2918 | 395,940 | na |
| 2010 | Soybean | 4-yr | Kruger 2918 | 395,940 | na |
| 2010 | Oat | 3-yr | IN09201 | na | 81 |
| 2010 | Oat | 4-yr | IN09201 | na | 81 |
| 2010 | Red clover | 3-yr | Medium | na | 13 |
| 2010 | Alfalfa | 4-yr | Freedom LH | na | 17 |
| 2011 | Maize | 2-yr | Agrigold 6395 BtRW | 79,530 | na |
| 2011 | Maize | 3-yr | Agrigold 6395 | 79,530 | na |
| 2011 | Maize | 4-yr | Agrigold 6395 | 79,530 | na |
| 2011 | Soybean | 2-yr | Kruger 287RR | 400,140 | na |
| 2011 | Soybean | 3-yr | Kruger 2918 | 400,140 | na |
| 2011 | Soybean | 4-yr | Kruger 2918 | 400,140 | na |
| 2011 | Oat | 3-yr | IN09201 | na | 81 |
| 2011 | Oat | 4-yr | IN09201 | na | 81 |
| 2011 | Red clover | 3-yr | Duration | na | 13 |
| 2011 | Alfalfa | 4-yr | Farm Science Genetics 400LH | na | 17 |
