# Supplementary material for: Increasing Cropping System Diversity Balances Productivity, Profitability and Environmental Health
Source: PLoS One. 2012 Oct 10;7(10):e47149. doi: 10.1371/journal.pone.0047149 (PMC3468434; doi:10.1371/journal.pone.0047149)
Supplement: Table S3 — Macronutrients applied in manufactured fertilizers, herbicide adjuvants, and manure in 2003–2011. Manufactured N, P, and K fertilizers were applied at rates that varied among years and rotations in response to soil test results. Manure was applied at a rate of 15.7 Mg ha−1 in maize phases of the 3-year and 4-year rotation systems, but moisture and nutrient concentrations varied among years, resulting in variable rates of macronutrient additions. (DOCX) [file pone.0047149.s004.docx]

Table S3. Macronutrients applied in manufactured fertilizers, herbicide adjuvants, and manure in 2003-2011.

|  |  |  | **Manufactured fertilizer^a^ (kg ha^‑1^)** | | | **Manure^b^ (kg ha^-1^)** | | |
| --- | --- | --- | --- | --- | --- | --- | --- | --- |
| **Year** | **Crop** | **Rotation** | **N** | **P** | **K** | **N** | **P** | **K** |
| 2003 | Maize | 2-yr | 153 | 0 | 0 | 0 | 0 | 0 |
| 2003 | Maize | 3-yr | 96 | 0 | 0 | 114 | 43 | 141 |
| 2003 | Maize | 4-yr | 57 | 0 | 0 | 114 | 43 | 141 |
| 2003 | Soybean | 2-yr | 0 | 0 | 0 | 0 | 0 | 0 |
| 2003 | Soybean | 3-yr | 0 | 0 | 0 | 0 | 0 | 0 |
| 2003 | Soybean | 4-yr | 0 | 0 | 0 | 0 | 0 | 0 |
| 2003 | Triticale/red clover | 3-yr | 28 | 0 | 0 | 0 | 0 | 0 |
| 2003 | Triticale/ alfalfa | 4-yr | 28 | 0 | 0 | 0 | 0 | 0 |
| 2003 | Alfalfa | 4-yr | 0 | 0 | 0 | 0 | 0 | 0 |
| 2004 | Maize | 2-yr | 112 | 0 | 0 | 0 | 0 | 0 |
| 2004 | Maize | 3-yr | 107 | 0 | 0 | 58 | 38 | 117 |
| 2004 | Maize | 4-yr | 70 | 0 | 0 | 58 | 38 | 117 |
| 2004 | Soybean | 2-yr | 2 | 0 | 0 | 0 | 0 | 0 |
| 2004 | Soybean | 3-yr | 1 | 0 | 0 | 0 | 0 | 0 |
| 2004 | Soybean | 4-yr | 1 | 0 | 0 | 0 | 0 | 0 |
| 2004 | Triticale/red clover | 3-yr | 28 | 0 | 0 | 0 | 0 | 0 |
| 2004 | Triticale/ alfalfa | 4-yr | 28 | 0 | 0 | 0 | 0 | 0 |
| 2004 | Alfalfa | 4-yr | 0 | 0 | 0 | 0 | 0 | 0 |
| 2005 | Maize | 2-yr | 114 | 0 | 0 | 0 | 0 | 0 |
| 2005 | Maize | 3-yr | 1 | 0 | 0 | 205 | 84 | 222 |
| 2005 | Maize | 4-yr | 1 | 0 | 0 | 205 | 84 | 222 |
| 2005 | Soybean | 2-yr | 1 | 0 | 0 | 0 | 0 | 0 |
| 2005 | Soybean | 3-yr | 1 | 0 | 0 | 0 | 0 | 0 |
| 2005 | Soybean | 4-yr | 1 | 0 | 0 | 0 | 0 | 0 |
| 2005 | Triticale/red clover | 3-yr | 28 | 0 | 0 | 0 | 0 | 0 |
| 2005 | Triticale/ alfalfa | 4-yr | 28 | 0 | 0 | 0 | 0 | 0 |
| 2005 | Alfalfa | 4-yr | 0 | 0 | 0 | 0 | 0 | 0 |
| 2006 | Maize | 2-yr | 158 | 20 | 112 | 0 | 0 | 0 |
| 2006 | Maize | 3-yr | 18 | 20 | 112 | 148 | 68 | 160 |
| 2006 | Maize | 4-yr | 18 | 20 | 112 | 148 | 68 | 160 |
| 2006 | Soybean | 2-yr | 19 | 20 | 112 | 0 | 0 | 0 |
| 2006 | Soybean | 3-yr | 19 | 20 | 112 | 0 | 0 | 0 |
| 2006 | Soybean | 4-yr | 19 | 20 | 112 | 0 | 0 | 0 |
| 2006 | Oat/red clover | 3-yr | 18 | 20 | 112 | 0 | 0 | 0 |
| 2006 | Oat/alfalfa | 4-yr | 18 | 20 | 112 | 0 | 0 | 0 |
| 2006 | Alfalfa | 4-yr | 18 | 20 | 112 | 0 | 0 | 0 |
| 2007 | Maize | 2-yr | 112 | 0 | 0 | 0 | 0 | 0 |
| 2007 | Maize | 3-yr | 0 | 0 | 0 | 121 | 70 | 94 |
| 2007 | Maize | 4-yr | 0 | 0 | 0 | 121 | 70 | 94 |
| 2007 | Soybean | 2-yr | 0 | 0 | 0 | 0 | 0 | 0 |
| 2007 | Soybean | 3-yr | 1 | 0 | 0 | 0 | 0 | 0 |
| 2007 | Soybean | 4-yr | 1 | 0 | 0 | 0 | 0 | 0 |
| 2007 | Oat/red clover | 3-yr | 0 | 0 | 0 | 0 | 0 | 0 |
| 2007 | Oat/alfalfa | 4-yr | 0 | 0 | 0 | 0 | 0 | 0 |
| 2007 | Alfalfa | 4-yr | 0 | 0 | 0 | 0 | 0 | 0 |
| 2008 | Maize | 2-yr | 213 | 20 | 49 | 0 | 0 | 0 |
| 2008 | Maize | 3-yr | 101 | 0 | 0 | 119 | 69 | 92 |
| 2008 | Maize | 4-yr | 101 | 20 | 49 | 119 | 69 | 92 |
| 2008 | Soybean | 2-yr | 1 | 20 | 49 | 0 | 0 | 0 |
| 2008 | Soybean | 3-yr | 1 | 0 | 0 | 0 | 0 | 0 |
| 2008 | Soybean | 4-yr | 1 | 20 | 49 | 0 | 0 | 0 |
| 2008 | Oat/red clover | 3-yr | 0 | 0 | 0 | 0 | 0 | 0 |
| 2008 | Oat/alfalfa | 4-yr | 0 | 20 | 49 | 0 | 0 | 0 |
| 2008 | Alfalfa | 4-yr | 0 | 20 | 49 | 0 | 0 | 0 |
| 2009 | Maize | 2-yr | 168 | 0 | 0 | 0 | 0 | 0 |
| 2009 | Maize | 3-yr | 0 | 0 | 0 | 122 | 52 | 113 |
| 2009 | Maize | 4-yr | 0 | 0 | 0 | 122 | 52 | 113 |
| 2009 | Soybean | 2-yr | 0 | 0 | 0 | 0 | 0 | 0 |
| 2009 | Soybean | 3-yr | 1 | 0 | 0 | 0 | 0 | 0 |
| 2009 | Soybean | 4-yr | 1 | 0 | 0 | 0 | 0 | 0 |
| 2009 | Oat/red clover | 3-yr | 0 | 0 | 0 | 0 | 0 | 0 |
| 2009 | Oat/alfalfa | 4-yr | 0 | 0 | 0 | 0 | 0 | 0 |
| 2009 | Alfalfa | 4-yr | 0 | 0 | 0 | 0 | 0 | 0 |
| 2010 | Maize | 2-yr | 175 | 59 | 178 | 0 | 0 | 0 |
| 2010 | Maize | 3-yr | 1 | 0 | 90 | 83 | 43 | 73 |
| 2010 | Maize | 4-yr | 1 | 0 | 178 | 83 | 43 | 73 |
| 2010 | Soybean | 2-yr | 1 | 59 | 178 | 0 | 0 | 0 |
| 2010 | Soybean | 3-yr | 0 | 0 | 90 | 0 | 0 | 0 |
| 2010 | Soybean | 4-yr | 0 | 0 | 178 | 0 | 0 | 0 |
| 2010 | Oat/red clover | 3-yr | 0 | 0 | 90 | 0 | 0 | 0 |
| 2010 | Oat/alfalfa | 4-yr | 0 | 0 | 178 | 0 | 0 | 0 |
| 2010 | Alfalfa | 4-yr | 0 | 0 | 178 | 0 | 0 | 0 |
| 2011 | Maize | 2-yr | 112 | 0 | 0 | 0 | 0 | 0 |
| 2011 | Maize | 3-yr | 1 | 0 | 0 | 106 | 52 | 82 |
| 2011 | Maize | 4-yr | 1 | 0 | 0 | 106 | 52 | 82 |
| 2011 | Soybean | 2-yr | 0 | 0 | 0 | 0 | 0 | 0 |
| 2011 | Soybean | 3-yr | 0 | 0 | 0 | 0 | 0 | 0 |
| 2011 | Soybean | 4-yr | 0 | 0 | 0 | 0 | 0 | 0 |
| 2011 | Oat/red clover | 3-yr | 0 | 0 | 0 | 0 | 0 | 0 |
| 2011 | Oat/alfalfa | 4-yr | 0 | 0 | 0 | 0 | 0 | 0 |
| 2011 | Alfalfa | 4-yr | 0 | 0 | 0 | 0 | 0 | 0 |

^a^ Manufactured N, P, and K fertilizers were applied at rates that varied among years and rotations in response to soil test results.

^b^ Manure was applied at a rate of 15.7 Mg ha^-1^ in maize phases of the 3-year and 4-year rotation systems, but moisture and nutrient concentrations varied among years, resulting in variable rates of macronutrient additions.
