# Supplementary material for: Increasing Cropping System Diversity Balances Productivity, Profitability and Environmental Health
Source: PLoS One. 2012 Oct 10;7(10):e47149. doi: 10.1371/journal.pone.0047149 (PMC3468434; doi:10.1371/journal.pone.0047149)
Supplement: Table S4 — Herbicide applications in 2003–2011 to maize and soybean in the three rotation systems. No herbicides were used for triticale, oat, red clover, and alfalfa grown within the 3-yr and 4-yr systems. Reported application rates reflect the effect of banding of herbicides over crop rows in the 3-yr and 4-yr systems. (DOCX) [file pone.0047149.s005.docx]

**Table S4.** Herbicide applications in 2003-2011 to maize and soybean in the three rotation systems.

| **Year** | **Crop** | **Rotation^a^** | **Active ingredient (ai)** | **Application (kg ai ha^-1^) ^b^** |
| --- | --- | --- | --- | --- |
| 2003 | Maize | 2-yr | S-metolachlor† | 1.600 |
| 2003 | Maize | 2-yr | Isoxaflutole‡ | 0.105 |
| 2003 | Maize | 2-yr | Nicosulfuron§ | 0.026 |
| 2003 | Maize | 2-yr | Rimsulfuron¶ | 0.013 |
| 2003 | Maize | 2-yr | Mesotrione# | 0.070 |
| 2003 | Maize | 3-yr | Nicosulfuron | 0.013 |
| 2003 | Maize | 3-yr | Rimsulfuron | 0.007 |
| 2003 | Maize | 3-yr | Mesotrione | 0.035 |
| 2003 | Maize | 4-yr | Nicosulfuron | 0.013 |
| 2003 | Maize | 4-yr | Rimsulfuron | 0.007 |
| 2003 | Maize | 4-yr | Mesotrione | 0.035 |
| 2003 | Soybean | 2-yr | S-metolachlor | 1.600 |
| 2003 | Soybean | 2-yr | Bentazon†† | 1.120 |
| 2003 | Soybean | 2-yr | Flumiclorac pentyl ester‡‡ | 0.060 |
| 2003 | Soybean | 2-yr | Clethodim§§ | 0.180 |
| 2003 | Soybean | 3-yr | S-metolachlor | 1.600 |
| 2003 | Soybean | 3-yr | Flumiclorac pentyl ester | 0.030 |
| 2003 | Soybean | 4-yr | S-metolachlor | 1.600 |
| 2003 | Soybean | 4-yr | Flumiclorac pentyl ester | 0.030 |
| 2004 | Maize | 2-yr | S-metolachlor | 1.600 |
| 2004 | Maize | 2-yr | Isoxaflutole | 0.105 |
| 2004 | Maize | 3-yr | Nicosulfuron | 0.013 |
| 2004 | Maize | 3-yr | Rimsulfuron | 0.007 |
| 2004 | Maize | 3-yr | Mesotrione | 0.047 |
| 2004 | Maize | 4-yr | Nicosulfuron | 0.013 |
| 2004 | Maize | 4-yr | Rimsulfuron | 0.007 |
| 2004 | Maize | 4-yr | Mesotrione | 0.047 |
| 2004 | Soybean | 2-yr | S-metolachlor | 1.600 |
| 2004 | Soybean | 2-yr | Bentazon | 1.120 |
| 2004 | Soybean | 2-yr | Clethodim | 0.105 |
| 2004 | Soybean | 3-yr | S-metolachlor | 1.600 |
| 2004 | Soybean | 3-yr | Bentazon | 0.560 |
| 2004 | Soybean | 4-yr | S-metolachlor | 1.600 |
| 2004 | Soybean | 4-yr | Bentazon | 0.560 |
| 2005 | Maize | 2-yr | S-metolachlor | 1.981 |
| 2005 | Maize | 2-yr | Isoxaflutole | 0.070 |
| 2005 | Maize | 2-yr | Nicosulfuron | 0.026 |
| 2005 | Maize | 2-yr | Rimsulfuron | 0.013 |
| 2005 | Maize | 3-yr | Nicosulfuron | 0.013 |
| 2005 | Maize | 3-yr | Rimsulfuron | 0.007 |
| 2005 | Maize | 3-yr | Mesotrione | 0.047 |
| 2005 | Maize | 4-yr | Nicosulfuron | 0.013 |
| 2005 | Maize | 4-yr | Rimsulfuron | 0.007 |
| 2005 | Maize | 4-yr | Mesotrione | 0.047 |
| 2005 | Soybean | 2-yr | S-metolachlor | 1.810 |
| 2005 | Soybean | 2-yr | Flumiclorac pentyl ester | 0.034 |
| 2005 | Soybean | 3-yr | S-metolachlor | 1.810 |
| 2005 | Soybean | 3-yr | Flumiclorac pentyl ester | 0.017 |
| 2005 | Soybean | 4-yr | S-metolachlor | 1.810 |
| 2005 | Soybean | 4-yr | Flumiclorac pentyl ester | 0.017 |
| 2006 | Maize | 2-yr | S-metolachlor | 1.981 |
| 2006 | Maize | 2-yr | Isoxaflutole | 0.088 |
| 2006 | Maize | 3-yr | Nicosulfuron | 0.013 |
| 2006 | Maize | 3-yr | Rimsulfuron | 0.007 |
| 2006 | Maize | 3-yr | Mesotrione | 0.053 |
| 2006 | Maize | 4-yr | Nicosulfuron | 0.013 |
| 2006 | Maize | 4-yr | Rimsulfuron | 0.007 |
| 2006 | Maize | 4-yr | Mesotrione | 0.053 |
| 2006 | Soybean | 2-yr | Glyphosate as isopropylamine salt¶¶ | 2.520 |
| 2006 | Soybean | 3-yr | Flumiclorac pentyl ester | 0.015 |
| 2006 | Soybean | 3-yr | Clethodim | 0.051 |
| 2006 | Soybean | 3-yr | Lactofen## | 0.053 |
| 2006 | Soybean | 4-yr | Flumiclorac pentyl ester | 0.015 |
| 2006 | Soybean | 4-yr | Clethodim | 0.051 |
| 2006 | Soybean | 4-yr | Lactofen | 0.053 |
| 2007 | Maize | 2-yr | S-metolachlor | 1.981 |
| 2007 | Maize | 2-yr | Isoxaflutole | 0.088 |
| 2007 | Maize | 3-yr | Nicosulfuron | 0.013 |
| 2007 | Maize | 3-yr | Rimsulfuron | 0.007 |
| 2007 | Maize | 3-yr | Mesotrione | 0.053 |
| 2007 | Maize | 4-yr | Nicosulfuron | 0.013 |
| 2007 | Maize | 4-yr | Rimsulfuron | 0.007 |
| 2007 | Maize | 4-yr | Mesotrione | 0.053 |
| 2007 | Soybean | 2-yr | Glyphosate as isopropylamine salt | 1.120 |
| 2007 | Soybean | 3-yr | Flumiclorac pentyl ester | 0.015 |
| 2007 | Soybean | 3-yr | Clethodim | 0.051 |
| 2007 | Soybean | 3-yr | Lactofen | 0.053 |
| 2007 | Soybean | 4-yr | Flumiclorac pentyl ester | 0.015 |
| 2007 | Soybean | 4-yr | Clethodim | 0.051 |
| 2007 | Soybean | 4-yr | Lactofen | 0.053 |
| 2008 | Maize | 2-yr | S-metolachlor | 1.981 |
| 2008 | Maize | 2-yr | Isoxaflutole | 0.088 |
| 2008 | Maize | 3-yr | Nicosulfuron | 0.013 |
| 2008 | Maize | 3-yr | Rimsulfuron | 0.007 |
| 2008 | Maize | 3-yr | Mesotrione | 0.053 |
| 2008 | Maize | 4-yr | Nicosulfuron | 0.013 |
| 2008 | Maize | 4-yr | Rimsulfuron | 0.007 |
| 2008 | Maize | 4-yr | Mesotrione | 0.053 |
| 2008 | Soybean | 2-yr | Glyphosate as isopropylamine salt | 1.120 |
| 2008 | Soybean | 3-yr | Flumiclorac pentyl ester | 0.015 |
| 2008 | Soybean | 3-yr | Clethodim | 0.051 |
| 2008 | Soybean | 3-yr | Lactofen | 0.053 |
| 2008 | Soybean | 4-yr | Flumiclorac pentyl ester | 0.015 |
| 2008 | Soybean | 4-yr | Clethodim | 0.051 |
| 2008 | Soybean | 4-yr | Lactofen | 0.053 |
| 2009 | Maize | 2-yr | S-metolachlor | 1.820 |
| 2009 | Maize | 2-yr | Isoxaflutole | 0.070 |
| 2009 | Maize | 3-yr | Nicosulfuron | 0.013 |
| 2009 | Maize | 3-yr | Rimsulfuron | 0.007 |
| 2009 | Maize | 3-yr | Mesotrione | 0.053 |
| 2009 | Maize | 4-yr | Nicosulfuron | 0.013 |
| 2009 | Maize | 4-yr | Rimsulfuron | 0.007 |
| 2009 | Maize | 4-yr | Mesotrione | 0.053 |
| 2009 | Soybean | 2-yr | Glyphosate as isopropylamine salt | 1.120 |
| 2009 | Soybean | 3-yr | Flumiclorac pentyl ester | 0.015 |
| 2009 | Soybean | 3-yr | Clethodim | 0.051 |
| 2009 | Soybean | 3-yr | Lactofen | 0.070 |
| 2009 | Soybean | 4-yr | Flumiclorac pentyl ester | 0.015 |
| 2009 | Soybean | 4-yr | Clethodim | 0.051 |
| 2009 | Soybean | 4-yr | Lactofen | 0.070 |
| 2010 | Maize | 2-yr | S-metolachlor | 1.820 |
| 2010 | Maize | 2-yr | Isoxaflutole | 0.070 |
| 2010 | Maize | 3-yr | Nicosulfuron | 0.013 |
| 2010 | Maize | 3-yr | Rimsulfuron | 0.007 |
| 2010 | Maize | 3-yr | Mesotrione | 0.053 |
| 2010 | Maize | 4-yr | Nicosulfuron | 0.013 |
| 2010 | Maize | 4-yr | Rimsulfuron | 0.007 |
| 2010 | Maize | 4-yr | Mesotrione | 0.053 |
| 2010 | Soybean | 2-yr | Glyphosate as isopropylamine salt | 1.400 |
| 2010 | Soybean | 3-yr | Flumiclorac pentyl ester | 0.023 |
| 2010 | Soybean | 3-yr | Clethodim | 0.051 |
| 2010 | Soybean | 3-yr | Lactofen | 0.088 |
| 2010 | Soybean | 4-yr | Flumiclorac pentyl ester | 0.023 |
| 2010 | Soybean | 4-yr | Clethodim | 0.051 |
| 2010 | Soybean | 4-yr | Lactofen | 0.088 |
| 2011 | Maize | 2-yr | S-metolachlor | 1.820 |
| 2011 | Maize | 2-yr | Isoxaflutole | 0.070 |
| 2011 | Maize | 3-yr | Nicosulfuron | 0.013 |
| 2011 | Maize | 3-yr | Rimsulfuron | 0.007 |
| 2011 | Maize | 3-yr | Mesotrione | 0.053 |
| 2011 | Maize | 4-yr | Nicosulfuron | 0.013 |
| 2011 | Maize | 4-yr | Rimsulfuron | 0.007 |
| 2011 | Maize | 4-yr | Mesotrione | 0.053 |
| 2011 | Soybean | 2-yr | Glyphosate as potassium salt | 1.203 |
| 2011 | Soybean | 2-yr | Glyphosate as isopropylamine salt | 1.050 |
| 2011 | Soybean | 3-yr | Flumiclorac pentyl ester | 0.015 |
| 2011 | Soybean | 3-yr | Clethodim | 0.051 |
| 2011 | Soybean | 3-yr | Lactofen | 0.070 |
| 2011 | Soybean | 4-yr | Flumiclorac pentyl ester | 0.015 |
| 2011 | Soybean | 4-yr | Clethodim | 0.051 |
| 2011 | Soybean | 4-yr | Lactofen | 0.070 |

^a^ No herbicides were used for triticale, oat, red clover, and alfalfa grown within the 3-yr and 4-yr systems.

^b^ Reported application rates reflect the effect of banding of herbicides over crop rows in the 3-yr and 4-yr systems.

† S-metolachlor: acetamide, 2-chloro-*N*-(2-ethyl-6-methylphenyl)-N-(2-methoxy-1-methylethyl)-,(S).

‡ Isoxaflutole: 5-cyclopropyl-4-(2-methylsulfonyl-4-trifl uoromethylbenzoyl) isoxazole.

§ Nicosulfuron: 2-((4,6-dimethoxypyrimidin-2-yl)aminocarbonyl) aminosulfonyl-N,N-dimethyl-3-pyridinecarboxamide.

¶ Rimsulfuron: N((4,6- dimethoxypyrimidin-2-yl)amino]carbonyl)-3-(ethylsulfonyl)-2-pyridine sulfonamide.

# Mesotrione: (2-(4-methylsulfonyl)-2-nitrobenzoyl)-1,3-cyclohexanedione.

†† Bentazon: 3-(1-methylethyl)-1H-2,1,3-benzothiadiazin-4(3H)-one 2,2-dioxide.

‡‡ Flumiclorac pentyl ester: (pentyl(2-chloro-4-fl uoro-5-(1,3,4,5,6,7-hexahydro-1,3-dioxo2H-isoindol-2-yl)phenoxy)acetate.

§§ Clethodim: (E)-2-(1-(((3-chloro-2-propenyl)oxy)imino)propyl)-5-(2-(ethylthio)propyl)-3-hydroxy-2-2cyclohenen-1-one.

¶¶ Glyphosate: N-(phosphonomethyl) glycine in the form of its isopropylamine or potassium salt.

## Lactofen: ethyl O{5-(2-chloro-a,a,a-trifluoro-p-toluoxy)-2-nitrobenzoyl}-DL-lactate.
