# Supplementary material for: Increasing Cropping System Diversity Balances Productivity, Profitability and Environmental Health
Source: PLoS One. 2012 Oct 10;7(10):e47149. doi: 10.1371/journal.pone.0047149 (PMC3468434; doi:10.1371/journal.pone.0047149)
Supplement: Table S5 — Simple and partial correlations between energy use within a given crop phase and mean rotation energy use and between energy use within a given crop phase and N fertilizer application rates. (DOCX) [file pone.0047149.s006.docx]

**Table S5.** Simple and partial correlations between energy use and N fertilizer application rates.

|  |  | Simple correlations^b^ | Partial correlations | |
| --- | --- | --- | --- | --- |
| Rotation length (yr) | Crop phase^a^ | r_EcErot_ | r_EcNc_ | pr_EcErot.Nc_ |
| 2 | m | 0.95***^c^ | 0.53** | 0.94*** |
| 2 | sb | 0.57** | 0.21 | 0.58** |
| 3 | m | 0.90*** | 0.82*** | 0.81*** |
| 3 | sb | 0.44** | 0.14 | 0.37* |
| 3 | sg | 0.69*** | 0.46** | 0.01 |
| 4 | m | 0.79*** | 0.71*** | 0.70*** |
| 4 | sb | 0.70*** | 0.35* | 0.54** |
| 4 | sg | 0.71*** | 0.49** | 0.66*** |
| 4 | a | 0.46** | -0.03 | 0.08 |

^a^ Abbreviations for crop phase: m = maize, sb = soybean, sg = small grain (triticale in 2003-2005, oat in 2006-2011), a = alfalfa.

^b^ Abbreviations for correlations: Ec = energy use within a crop phase, Erot = mean rotation energy use, Nc = N application rate within a given crop phase. The term pr_EcErot.Nc_ denotes the partial correlation between Ec and Erot, partialling out variation due to Nc.

^c^ The symbols *, ** and *** denote significant correlations at P < 0.01, 0.001 and 0.0001, respectively.
