# Supplementary material for: Increasing Cropping System Diversity Balances Productivity, Profitability and Environmental Health
Source: PLoS One. 2012 Oct 10;7(10):e47149. doi: 10.1371/journal.pone.0047149 (PMC3468434; doi:10.1371/journal.pone.0047149)
Supplement: Text S1 — Detailed description of experimental site, management practices, scientific methods and statistical approach. (DOCX) [file pone.0047149.s007.docx]

**Supporting Information**

**Text S1. Detailed Methods**

***Experimental site and management* *practices***

The experiment was conducted at the Iowa State University Marsden Farm in Boone, Co., Iowa, USA (42^°^01' N; 93^°^47' W; 333 m above sea level), on Clarion loam (fine-loamy, mixed, superactive, mesic, Typic Hapludolls), Nicollet loam (fine-loamy, mixed, superactive, mesic, Aquic Hapludolls), and Webster silty clay loam (fine-loamy, mixed, superactive, mesic, Typic Endoaquolls) soils. The entire site was planted with oat in 2001 and experimental plots were established in 2002 using a randomized complete block design, with each crop phase of each rotation system present every year in four replicate blocks (Fig. S1). Plot size was 18 m x 85 m. Prior to the initiation of the experiment, the site had been managed with a maize/soybean rotation receiving conventional fertilizer and herbicide inputs. Soil samples taken to a depth of 20 cm on 8 Nov 2002 indicated a mean buffer pH of 6.8, a mean organic matter concentration (via combustion analysis) of 51 g kg^-1^, a mean P concentration (via the Bray-1 procedure) of 31 g kg^-1^, and a mean K concentration (via ammonium acetate extraction) of 167 g kg^-1^. Temperature conditions during the experiment did not deviate greatly from the long-term mean, but precipitation was generally greater than the long-term mean, with 2008 and 2010 being much wetter than average (Table S1). Several plots were flooded for up to three or four days in 2008 and 2010, but in general, crop growth conditions during the period of the experiment were good to excellent.

Three rotation systems were compared in the experiment: a 2-yr maize-soybean system; a 3-yr maize-soybean-small grain/red clover system; and a 4-yr maize-soybean-small grain/alfalfa-alfalfa system. Crop identities and seeding rates are shown in Table S2. A non-genetically engineered soybean variety was used in all systems in 2003-2005. However, beginning in 2006 and continuing though 2011, a transgenic glyphosate-tolerant soybean variety was used in the 2-yr rotation, whereas a non-genetically engineered variety was used in the 3-yr and 4-yr rotations. Similarly, a non-genetically engineered maize hybrid was used in all systems in 2003-2007, but in 2008-2011, a hybrid with transgenic resistance to insect pests was used in the 2-yr rotation. A near-isogenic maize hybrid without transgenes was used in 2008-2011 in the 3-yr and 4-yr rotations. Triticale was used as the small grain crop in 2003-2005, whereas oat was used in 2006-2011. Triticale and oat straw was baled and removed after grain harvest. Red clover and alfalfa were planted with triticale or oat. Red clover was not harvested for forage, but was plowed into the soil as a green manure crop. Seeding year alfalfa was not harvested in 2003 (due to dry conditions that limited growth), but was harvested once in 2004-2011. Established second-year alfalfa was harvested three times or four times, depending on moisture conditions present in each year, before being plowed in late November or early December.

Tillage regimes differed among rotation systems. In the 2-yr rotation, a combination of fall chisel plowing and spring field cultivation was used between maize harvest and soybean planting; spring field cultivation was used between soybean harvest and maize planting. In the 3-yr system, a combination of fall chisel plowing and spring field cultivation was used between maize harvest and soybean planting; zero tillage or spring disking was used between soybean harvest and the planting of small grains (triticale or oat) and red clover; and fall moldboard plowing followed by spring disking and field cultivation were used to incorporate clover sod and prepare a seedbed for maize. Tillage practices in the 4-yr system were the same as in the 3-yr system, except for a longer period without soil disturbance, from small grain and alfalfa establishment until alfalfa sod was fall moldboard plowed.

Synthetic fertilizers were applied in the 2-yr rotation at conventional rates based on soil tests, whereas composted cattle manure and reduced rates of synthetic fertilizers were applied in the 3-yr and 4-yr rotations (Table S3). The late spring nitrate test was used for maize in each rotation system to determine treatment-specific rates for post-emergence side-dress N applications (1). Composted manure was applied in October of each year at a rate of 15.7 Mg ha^-1^ (fresh weight basis) to plots of red clover in the 3-yr rotation and to plots of established alfalfa in the 4-yr rotation. This corresponded to a mean dry matter application rate of 8.3 Mg ha^-1^. Calculated application rates of total N, P, and K in composted manure, reflecting analyses conducted by the Iowa State University Soil and Plant Analysis Laboratory, are shown in Table S3.

Weed management in the 2-yr rotation was based largely on herbicides applied at conventional rates (Table S4). Lower quantities of herbicides were applied in the 3-yr and 4-yr rotations due to application of herbicides in 38-cm-wide bands over maize and soybean rows rather than broadcast spraying, interrow cultivation in maize and soybean, and elimination of herbicide applications in the small grain and forage legume crops (Table S4). Insecticides were applied in all soybean plots for soybean aphid control (*Aphis glycines*) in three years of the study. Lambda-cyhalothrin was applied at 0.035 kg active ingredient ha^-1^ in 2007 and at 0.029 kg ai ha^-1^ in 2008; chlorpyrifos was applied at 0.560 kg ai ha^-1^ in 2009.

***Crop sampling***

Yields of maize and soybean were determined from sample areas of 382 to 765 m^2^, compromising 6 to 12 rows in the central area of each plot, using a combine harvester and a weigh wagon. Triticale and oat grain yields were determined in the same way from whole plots (1530 m^2^). Yields of triticale and oat straw and alfalfa hay were determined by weighing bales harvested from whole plots. After determining crop moisture concentrations, yields were adjusted to moisture levels of 155 g kg^-1^ for maize, 130 g kg^-1^ for soybean, 135 g kg^-1^ for triticale grain, 140 g kg^-1^ for oat grain, 100 g kg^-1^ for straw, and 150 g kg^-1^ for alfalfa hay.

***Weed sampling***

Ambient weed biomass was determined in maize and soybean plots by clipping all weed shoot material from eight 3.05-m x 0.76-m sampling areas in each plot (18.5 m^2^ plot^-1^) in September or early October each year. Weed biomass in small grain stubble with red clover, small grain stubble with alfalfa, and second year alfalfa was determined by clipping all weed shoot material in eight 0.25-m^2^ quadrats in each plot (2.0 m^2^ plot^-1^) in September or early October, and then drying and weighing the samples.

To study weed seed bank dynamics, we collected 20 32-mm-diameter soil cores to a depth of 20 cm from each plot in October of each year. These cores were composited by plot and a 640-cm^3^ subsample was drawn from each sample and washed in an elutriator to separate weed seeds from soil (2). Weed seeds were enumerated by species and analyzed for viability using direct germination and tetrazolium tests (3).

***Energy use***

Energy inputs were divided into five categories: seed, fertilizer, pesticides, fuel for field operations, and propane and electricity used for drying maize grain after harvest. Data were obtained from logs describing all field operations, material inputs, and crop moisture characteristics for the experimental plots during the study period.

There is limited information on fossil energy consumption in the production of seeds used for planting. For seeds of hybrid maize, we used the value reported by Graboski (2002) (3) (10.0 MJ kg^-1^). For seeds of all other crops in our study (soybean, triticale, oat, red clover, and alfalfa), we used the value reported by Sheehan et al. (4) for soybean (4.7 MJ kg^-1^).

Energy costs of manufactured fertilizers were evaluated on the basis of the mass of the elements applied using data from Shapouri et al. (5) (N: 56.9 MJ kg^-1^; P: 9.3 MJ kg^-1^; K: 7.0 MJ kg^-1^). Energy costs for herbicides and insecticides were evaluated on the basis of the mass of active ingredients applied and their formulation as commercial products using data from Bhat et al. 1994 (6) (herbicides: 235 MJ kg^-1^; insecticides: 265 MJ kg^-1^). Values for diesel fuel consumed during field operations were obtained from Hanna (7); for operations not specifically listed, the closest equivalent was used as a substitute. The energy content of diesel fuel was assumed to be 42.5 MJ L^-1^ (5).

Maize grain is often harvested at a moisture content that exceeds that which is desirable for storage of the grain. Consequently, energy in the forms of propane and electricity is used to dry it. We assumed an energy cost for grain drying of 67.2 MJ Mg^-1^ for each percentage point of moisture removed (8). The necessary reduction in moisture was calculated as the difference between the moisture content of grain harvested from the field plots and the target moisture content of 15.5%. Maize harvested below 15.5% moisture content was assumed to not require artificial drying.

***Economic analyses***

Labor requirements, costs, and returns for the different rotation systems were assessed using data from several sources. Labor times for machinery operations were based on values reported by Hanna (9) and costs for labor and machinery wear and maintenance were assigned based on data from Duffy (10). Costs for manufactured fertilizers, seeds, pesticides, interest on loans, crop insurance, and miscellaneous items were estimated using data from Duffy (10) and local agricultural dealers. We assumed manure was generated by on-farm livestock and therefore without cost for the material, but with labor and machinery costs for spreading based on data from (8) and (9). Iowa market year crop prices were obtained from the USDA National Agricultural Statistics Service (11), and gross revenue was calculated for each plot in each year as the product of crop price and yield.

We placed primary emphasis on determining the economic performance characteristics of whole rotation systems under contrasting management strategies, rather than the economic performance of individual crop phases within the different rotations. Consequently, we evaluated net returns to land and management on a unit land area basis, with land units divided in two equal portions for maize and soybean in the 2-yr rotation; three equal portions for maize, soybean, and small grains with red clover in the 3-yr rotation; and four equal portions for maize, soybean, small grains with alfalfa, and alfalfa in the 4-yr rotation. Net returns to land and management represented returns to a farm operation calculated without accounting for costs of land (e.g., rent or mortgage payments), management time (e.g., marketing), or possible federal subsidies.

***References for SI***

1. Blackmer, A.M., R.D. Voss, and A.P. Mallarino. 1997. Nitrogen fertilizer recommendations for corn in Iowa. PM-1714. (Iowa State University Extension and Outreach, Ames, IA.) On-line at: http://www.extension.iastate.edu/ Publications/PM1714.pdf. Last accessed on May 17, 2012.

2. Wiles, L.J., D.H. Barlin, E.E. Schweitzer, H.R. Duke, and D.E. Whitt. 1996. A new soil sampler and elutriator for collecting and extracting weed seeds from soil. *Weed Tech* 10:35-41.

3. Graboski, M.S. 2002. Fossil energy use in the manufacture of corn ethanol. National Corn Growers Association, St. Louis, MO. On-line at: http://www.oregon.gov/ENERGY/RENEW/Biomass/docs/FORUM/FossilEnergyUse.pdf?ga=t. Last accessed on May 17, 2012.

4. International Seed Testing Association. 1985. International rules for seed testing. *Seed Sci Tech* 13:300–520.

5. Sheehan, J., Camobreco, V., Duffield, J., Graboski, M. & Shapouri, H. 1998. Life cycle inventory of biodiesel and petroleum diesel for use in an urban bus. Publ. No. SR-580-24089. (U.S. Department of Energy-National Renewable Energy Laboratory, Golden, CO) On-line at: <http://www.nrel.gov/docs/legosti/> fy98/24089.pdf. Last accessed on May 17, 2012.

6. Shapouri, H., P.W. Gallagher, W. Nefstead, R. Schwartz, S. Noe, and R. Conway. 2010. 2008 Energy balance for the corn-ethanol industry. Agricultural Economic Report No. 846. (U.S. Department of Agriculture, Washington, DC.) On-line at: http://www.usda.gov/oce/reports/energy/ 2008Ethanol_June_final.pdf. Last accessed on May 17, 2012.

7. Bhat, M.G., B.C. English, A.F. Turhollow, and H. O. Nyangito. 1994. Energy in synthetic fertilizers and pesticides: revisted. (Oak Ridge National Laboratory, U.S. Department of Energy, Oak Ridge, TN).

8. Hanna, M. 2005. Fuel required for field operations. Ag Decision Maker File A3-27. (Iowa State University Outreach and Extension, Ames, IA.) On-line at: http://www.extension.iastate.edu/agdm/crops/html/a3-27.html. Last accessed on May 17, 2012.

9. Edwards, W. 2009. Comparison of drying systems. Ag Decision Maker File A1-20. (Iowa State University Extension and Outreach, Ames, IA) On-line at: http://www.extension.iastate.edu/agdm/crops/xls/a1-20dryingcostcalculator.xls. Last accessed on May 17, 2012.

10. Hanna, M. 2001. Estimating field capacity of farm machines. PM-696. (Iowa State University Outreach and Extension, Ames, IA) On-line at: http://www.extension.iastate.edu/Publications/PM696.pdf. Last accessed on May 17, 2012.

11. Duffy, M. 2012. Estimated costs of crop production in Iowa, 2000-2012. (Iowa State University Extension and Outreach, Ames, IA) On-line at: http://www.extension.iastate.edu/agdm/crops/html/a1-20.html. Last accessed on May 17, 2012.

12. National Agricultural Statistics Service (NASS). 2012. Crop values annual summary. NASS, U.S. Department of Agriculture, Washington, DC. On-line at: http://usda.mannlib.cornell.edu/MannUsda/viewDocumentInfo.do ?documentID=1050. Last accessed on May 17, 2012.
